# Belowground Phytolith-Occluded Carbon of Monopodial Bamboo in China: An Overlooked Carbon Stock

**DOI:** 10.3389/fpls.2018.01615

**Published:** 2018-11-06

**Authors:** Chen Chen, Zhangting Huang, Peikun Jiang, Junhui Chen, Jiasen Wu

**Affiliations:** ^1^State Key Laboratory of Subtropical Silviculture, Zhejiang A&F University, Lin’an, China; ^2^School of Environmental and Resource Sciences, Zhejiang A&F University, Lin’an, China; ^3^Zhejiang Provincial Collaborative Innovation Center for Bamboo Resources and High-efficiency Utilization, Lin’an, China

**Keywords:** PhytOC, aboveground biomass, belowground trunk and rhizome, carbon sequestration, phytolith

## Abstract

Phytolith-occluded carbon (PhytOC), a highly stable carbon (C) fraction resistant to decomposition, plays an important role in long-term global C sequestration. Previous studies have demonstrated that bamboo plants contribute greatly to PhytOC sink in forests based on their aboveground biomass. However, little is known about the contribution of belowground parts of bamboo to the PhytOC stock. Here, we reported the phytolith and PhytOC accumulation in belowground trunk and rhizome of eight monopodial bamboo species that widely distributed across China. The results showed that the belowground parts made up an average of 39.41% of the total plant biomass of the eight bamboo species. There were significant (*p* < 0.05) variations in the phytolith and PhytOC concentrations in the belowground trunk and rhizome between the bamboo species. The mean concentrations of PhytOC in dry biomass ranged from 0.34 to 0.83 g kg^-1^ in the belowground rhizome and from 0.10 to 0.94 g kg^-1^ in the belowground trunk across the eight bamboo species, respectively. The mean PhytOC stocks in belowground biomass ranged from 2.57 to 23.71 kg ha^-1^, occupying an average of 23.36% of the total plant PhytOC stocks. This implies that 1.01 × 10^5^ t PhytOC was overlooked based on the distribution of monopodial bamboos across China. Therefore, our results suggest that the belowground biomass of bamboo represents an important PhytOC stock, and should be taken into account in future studies in order to better quantifying PhytOC sequestration capacity.

## Introduction

Increased greenhouse gas (GHG) emissions have been widely accepted as the main cause of climate change, which threatens the sustainability of terrestrial ecosystem (Kosten et al., 2010; IPCC, 2014). Among the GHGs, the CO_2_ emission rate had increased to 3.11 × 10^11^ Mg per year by 2010 at the global scale (DOE, 2008). Methods that can reduce the speed of rapidly rising CO_2_ concentrations are urgently needed to contribute to climate change mitigation. Terrestrial biogeochemical carbon (C) sequestration is one of the most promising approaches for long-term atmospheric CO_2_ sequestration (IPCC, 2014).

Occlusion of C within phytoliths (PhytOC) as an effective mechanism of biotic C sequestration has received much attention in recent years (Parr and Sullivan, 2005; Song et al., 2012a,b, 2016; Yang et al., 2018). Phytolith, also referred to as plant opal, is an amorphous silica that formed in living plants (Wang and Lü, 1993; Parr and Sullivan, 2005). During the formation of phytolith, some organic C can be occluded in plant tissues. Previous studies demonstrated that PhytOC is highly stable and could be preserved in the soil for several 1000s of years after plant decomposition (Wilding et al., 1967; Parr and Sullivan, 2005; Santos et al., 2010). For example, Parr and Sullivan (2005) found that PhytOC could contribute up to 82% of the total soil C pool after 2000 years decomposition in Numundo oil palm (*Elaeis guineensis*) plantations. It is also suggested that PhytOC makes up between 15 and 37% of the estimated global accumulation rate (24 kg C ha^-1^ yr^-1^) of stable soil C, demonstrating the significant potential of PhytOC in the long-term terrestrial C sequestration (Parr and Sullivan, 2005; Song et al., 2012a).

The PhytOC concentrations in different plants vary greatly due to their differences in the capacity for phytolith accumulation (Parr et al., 2010; Song et al., 2012b, 2017; Yang et al., 2015; Xiang et al., 2016). Bamboo, a typical phytolith-accumulator (Parr et al., 2010), has been shown to have a greater production of PhytOC in comparison with other plants such as sugarcane (Parr et al., 2009), rice (Li et al., 2013), and millet (Zuo and Lü, 2011). Being predominantly distributed in the tropical and subtropical regions, bamboo has a global area of 2.2 × 10^7^ ha by 2010 and is increasing at a rate of 3% annually (Cao et al., 2011; Zhou et al., 2011). It has been estimated that the present annual PhytOC sink in China’s forests is 1.7 ± 0.4 Tg CO_2_ yr^-1^, 30% of which is contributed by bamboo because the production flux of PhytOC through tree leaf litter for bamboo is 3–80 times higher than that of other forest types (Song et al., 2013).

The potential of PhytOC sequestration in bamboo species also varies depending on the rhizomatous forms (Li et al., 2014a; Xiang et al., 2016). Monopodial scattering bamboo (typically Moso bamboo and Lei bamboo) forests accounted for 77.71% of the total area of bamboo forests in China and were estimated to contribute 75% of the total PhytOC sequestration in Chinese bamboo (Li et al., 2014b). Yang et al. (2015) further demonstrated that the PhytOC production flux contributed by aboveground biomass (including branches and culms) was 1.18 to 1.78 times compared with those estimated by leaf samples for eight monopodial bamboo species due to their larger biomass. Although existing research suggest the significant role of global PhytOC sequestration through bamboo plants, their estimates were only based on the aboveground biomass and the contribution of belowground parts was never determined. Bamboo plants usually have vigorous rhizomes with high biomass. For example, the belowground biomass of Moso bamboo could account for more than one third of total stand biomass (Wang et al., 2013). Given the large phytolith accumulation in bamboo branches and culm in our previous studies (Huang et al., 2014; Yang et al., 2015), we infer that the phytolith could also be accumulated in the bamboo rhizomes and a large amount of PhytOC sequestered in the belowground biomass may have been overlooked in previous studies, leading to a severe underestimation of the PhytOC stock in bamboo forests. The purposes of this study are (1) to examine and compare the concentration of phytolith and PhytOC in belowground trunk and rhizome and (2) to estimate the PhytOC stocks in belowground biomass of bamboo species that widely distributed across China. We hypothesize that the bamboo species differ in PhytOC concentrations in their belowground trunks and rhizomes, and that the belowground parts make a significant contribution to the total PhytOC sequestration of bamboo plants.

## Materials and Methods

### Experimental Site and Sampling

We selected eight monopodial bamboo species that account for more than 85% of the total area of monopodial bamboo forests in Zhejiang and Anhui Provinces, China. The eight bamboo species are *Phyllostachys heterocycla* (Carr.) Mitford ‘Pubescens’ (PHMP), *Phyllostachys praecox* C. D. Chu ‘Prevernalis’ (PPP), *Phyllostachys prominens* W. Y. Xiong (PP), *Pseudosasa amabilis* (McClure) Keng f (PAMK), *Phyllostachys glauca* McClure (PGM), *Pleioblastus amarus* (Keng) Keng f (PAKK)*, Phyllostachys heteroclada* Oliver (PHO), and *Bambusa piscatorum* McClure (BPM). Detailed sampling site information is given in Table 1. For each species, four plots with an area size of 20 m × 20 m were established in the bamboo forest. The plots in each forest had similar site conditions including elevation, soil type, slope gradient and aspect. The average diameter at breast height (DBH) and stem density were determined. In each plot, one individual bamboo plant having an DBH similar to the mean values was selected and used to determine the biomass of organs including leaves, branches, culms and belowground trunk. The silicon content, phytolith content, C content of phytolith, and PhytOC content per dry biomass were also determined. The rhizome for each species was collected from four subplots of 1 m × 1 m randomly established in each plot. All leaves, branches, and culms of each sample plant, and the belowground trunk and rhizome were weighed separately.

**Table 1:** Site information of sampling plots of eight monopodial bamboo species studied.

Bamboo species	Abbreviations	Sampling site	Longitude and latitude	Altitude (m)	Density (plants⋅ha^-1^)	DBH^a^ (cm)	Parent material
*Phyllostachys heterocycla* (Carr.)	PHMP	Hangzhou, Zhejiang	N 30°14′22″	149.18	2200	9.60	Tuff
Mitford ‘Pubescens’			E 119°2′30″				
*Phyllostachys praecox* C. D. Chu	PPP	Lin’an, Zhejiang	N 30°14′	150.00	20450	3.90	Arenaceous shale
‘Prevernalis’			E 119°42′				
*Phyllostachys prominens* W. Y.	PP	Tonglu, Zhejiang	N 29°48′0″	208.35	9700	6.70	Tuff
Xiong			E 119°34′24″				
*Pseudosasa amabilis* (McClure)	PAMK	Lin’an, Zhejiang	N 30°15′43″	50.10	36875	2.40	Tuff
Keng f			E 119°43′38″				
*Phyllostachys glauca* McClure	PGM	Ningguo, Anhui	N 30°29′24″	101.50	28150	2.85	Arenaceous shale
			E 119°9′1″				
*Pleioblastus amarus* (Keng) Keng f	PAKK	Lin’an, Zhejiang	N 30°11′31″	150.60	78400	2.50	Tuff
			E 119°51′1″				
*Phyllostachys heteroclada* Oliver	PHO	Lin’an, Zhejiang	N 30°18′55″	553.63	52500	2.15	Tuff
			E 119°27′9″				
*Bambusa piscatorum* McClure	BPM	Lin’an, Zhejiang	N 30°19′6″	425.88	45625	2.10	Tuff
			E 119°27′20″				


*^*a*^DBH, diameter at breast height. PHMP, *Phyllostachys heterocycla* (Carr.) Mitford ‘Pubescens’; PPP, *Phyllostachys praecox* C. D. Chu ‘Prevernalis’; PP, *Phyllostachys prominens* W. Y. Xiong; PAMK, *Pseudosasa amabilis* (McClure) Keng f; PGM, *Phyllostachys glauca* McClure; PAKK, *Pleioblastus amarus* (Keng) Keng f; PHO, *Phyllostachys heteroclada* Oliver; BPM, *Bambusa piscatorum* McClure.*

### Sample Measurements

Each sample was mixed, rinsed with ultrapure water and ultrasonic cleaning to clear all clays contaminated on the bamboo roots. The plant samples were oven-dried at 70°C for 48 h to a constant mass and then ground to pass through a 0.25-mm sieve for chemical analysis. Phytoliths in samples were extracted using a microwave digestion method (Parr et al., 2001). The phytolith extracts were transferred into pre-weighed centrifugal tubes, dried at 65°C for 48 h in an oven, and then weighed. A K_2_Cr_2_O_7_ solution (0.8 M) was used to detect whether the organic matter surrounding the phytolith had been completely removed before the determination of PhytOC (Parr et al., 2010). The PhytOC was determined according to the PhytOC alkali spectrophotometry method (Yang et al., 2014). The accuracy and repeatability of this analytical method was well verified against the results obtained with acid dissolution-Elementar Vario MAX CN method (Germany) (Yang et al., 2014). In this method, a 0.01 g phytolith sample was placed into a 10 mL centrifuge tube, 0.5 mL 10 M NaOH added, and incubated for 12 h at 25°C to dissolve the phytoliths. The extract was further treated with 1.0 mL of 0.8 M K_2_Cr_2_O_7_ solution followed by addition of 4.6 mL of concentrated H_2_SO_4_ to oxidize the released organic C. The obtained solutions were placed in a water bath at 98°C for 1 h, and the concentration of PhytOC in the solutions was determined colorimetrically at 590 nm on a Hitachi 150-20 spectrophotometer (Hitachi, Ltd., Tokyo, Japan).

### Data Calculation and Statistical Analysis

C concentration in phytolith, PhytOC concentration in dry biomass and PhytOC stock were calculated using the following formulas:

(1) $$C\;\mathrm{concentration}\;\mathrm{in}\;\mathrm{phytolith}\;(g\;{\mathrm{kg}}^{-1})=C\;\mathrm{content}\;\mathrm{in}\;\mathrm{phytolith}\;(g)/\mathrm{phytolith}\;\mathrm{weight}\;(\mathrm{kg})$$

(2) $$\mathrm{PhytOC}\;\mathrm{concentration}\;(g\;{\mathrm{kg}}^{-1})=C\;\mathrm{content}\;\mathrm{in}\;\mathrm{phytolith}\;(g)/\mathrm{dry}\;\mathrm{biomass}\;\mathrm{(kg)}$$

(3) $$\mathrm{PhytOC}\;\mathrm{stock}\;(\mathrm{kg}\;{\mathrm{ha}}^{-1})=\sum \left[\mathrm{PhytOC}\;\mathrm{concentration}\;(g\;{\mathrm{kg}}^{-1})\;\times \;\mathrm{biomass}\;(\mathrm{kg}\;{\mathrm{ha}}^{-1})\;\times \;10^{-3}\right]$$

MS Excel 2010 and SPSS 18 software were used to carry out data processing and statistical analysis. One-way ANOVA followed by LSD test (*p* < 0.05) were used to examine the difference in phytolith and PhytOC contents among different plant species.

## Results

### Belowground Biomass of Eight Monopodial Bamboo Species

The total aboveground biomass (including leaves, branches, and culm) ranged from 20.82 to 48.68 t ha^-1^ per dry weight across the eight species, with the highest in PAKK [*Pleioblastus amarus* (Keng) Keng f] and lowest in PP (*Phyllostachys prominens* W. Y. Xiong) (Table 2). The biomass of the rhizome was much smaller than that of the aboveground across the eight species with the exception that the biomass of rhizome of PP was almost three times higher than that in the aboveground. The biomass of the belowground trunk ranged from 1.19 to 7.18 t ha^-1^ across the eight species. The proportion of belowground biomass to total biomass varied from 18.64% for PHO (*Phyllostachys heteroclada* Oliver) to 74.91% for PP, with a mean of 39.41%.

**Table 2:** Biomass of eight monopodial bamboo species studied.

Bamboo species	Aboveground biomass (t ha^-1^)^a^	Biomass of rhizome (t ha^-1^)	Biomass of belowground trunk (t ha^-1^)	Belowground biomass (t ha^-1^)	Ratio of belowground to total biomass (%)
PHMP	45.94	22.19	2.17	24.36	34.65
PPP	24.47	19.78	2.96	22.74	48.17
PP	20.82	60.71	1.45	62.16	74.91
PAMK	35.55	13.23	1.33	14.55	29.05
PGM	37.85	25.65	1.89	27.54	42.11
PAKK	48.68	29.68	7.18	36.86	43.09
PHO	25.24	4.28	1.50	5.78	18.64
BPM	24.52	6.83	1.19	8.03	24.66


*^*a*^Data were cited from Huang (2014) and Yang (2016). The aboveground biomass includes leaves, branches and culm. PHMP, *Phyllostachys heterocycla* (Carr.) Mitford ‘Pubescens’; PPP, *Phyllostachys praecox* C. D. Chu ‘Prevernalis’; PP, *Phyllostachys prominens* W. Y. Xiong; PAMK, *Pseudosasa amabilis* (McClure) Keng f; PGM, *Phyllostachys glauca* McClure; PAKK, *Pleioblastus amarus* (Keng) Keng f; PHO, *Phyllostachys heteroclada* Oliver; BPM, *Bambusa piscatorum* McClure.*

### Phytolith and PhytOC Concentrations of Bamboo in Belowground Biomass

There was a significant (*p* < 0.05) variation in the concentrations of Si, phytolith, C concentration in phytolith, and PhytOC in belowground biomass among the eight bamboo species (Table 3). The concentration of Si and phytolith in the rhizome ranged from 8.43 g kg^-1^ for PHMP [*Phyllostachys heterocycla (Carr*.) *Mitford‘Pubescens’*] to 21.05 g kg^-1^ for PGM (*Phyllostachys glauca McClure*), and from 11.20 g kg^-1^ for PAKK to 35.44 g kg^-1^ for PGM, respectively. The C concentration in phytolith in the rhizome ranged from 11.02 g kg^-1^ for BPM (*Bambusa piscatorum McClure*) to 80.42 g kg^-1^ for PHMP. There were no significant differences in the C concentration in phytolith in the rhizome among the other seven bamboo species except PHMP.

**Table 3:** The concentrations of Si and phytolith, C concentration in phytolith and PhytOC/dry biomass in the belowground trunk and rhizome of eight monopodial bamboo species.

Organ	Bamboo species	Si (g⋅kg^-1^)	Phytolith (g⋅kg^-1^)	C concentration in phytolith (g⋅kg^-1^)	PhytOC/dry biomass (g⋅kg^-1^)
Rhizome	PHMP	8.43 ± 3.53b	14.69 ± 3.12bc	80.42 ± 16.87a	0.83 ± 0.38a
	PPP	14.51 ± 2.47ab	34.93 ± 7.39a	32.27 ± 19.17b	0.83 ± 0.54a
	PP	10.58 ± 5.83b	24.53 ± 9.55ab	28.33 ± 2.52b	0.38 ± 0.06a
	PAMK	16.16 ± 9.17ab	19.78 ± 3.96bc	34.63 ± 27.20b	0.67 ± 0.43a
	PGM	21.05 ± 15.00a	35.44 ± 17.84a	23.20 ± 19.70b	0.66 ± 0.50a
	PAKK	10.47 ± 3.93b	11.20 ± 5.33c	32.14 ± 2.75b	0.58 ± 0.08a
	PHO	10.70 ± 2.90ab	20.61 ± 5.57bc	23.94 ± 18.32b	0.53 ± 0.34a
	RMBPM	13.90 ± 4.69ab	34.74 ± 9.77a	11.02 ± 2.21b	0.34 ± 0.04a
Belowground trunk	PHMP	4.88 ± 3.75bc	10.82 ± 0.78ab	57.25 ± 23.53bcd	0.31 ± 0.05bcd
	PPP	2.30 ± 0.60c	10.68 ± 1.58ab	179.99 ± 40.04a	0.94 ± 0.53a
	PP	3.87 ± 1.03bc	6.13 ± 0.66c	77.71 ± 67.83bcd	0.10 ± 0.02d
	PAMK	5.62 ± 2.25b	11.95 ± 4.57ab	38.51 ± 35.06cd	0.19 ± 0.08d
	PGM	5.12 ± 1.23bc	9.91 ± 5.45bc	138.58 ± 139.21ab	0.25 ± 0.04cd
	PAKK	6.89 ± 1.70b	9.10 ± 3.46bc	132.66 ± 82.84abc	0.54 ± 0.16bc
	PHO	14.07 ± 3.67a	14.95 ± 2.79a	40.73 ± 18.44cd	0.61 ± 0.13b
	RMBPM	5.05 ± 1.34bc	5.88 ± 1.19c	23.44 ± 5.57d	0.21 ± 0.06d


*Values are means ± standard error of four replicates. Means followed by different letters within a column are significantly different at the *p* < 0.05 level. PHMP, *Phyllostachys heterocycla* (Carr.) Mitford ‘Pubescens’; PPP, *Phyllostachys praecox* C. D. Chu ‘Prevernalis’; PP, *Phyllostachys prominens* W. Y. Xiong; PAMK, *Pseudosasa amabilis* (McClure) Keng f; PGM, *Phyllostachys glauca* McClure; PAKK, *Pleioblastus amarus* (Keng) Keng f; PHO, *Phyllostachys heteroclada* Oliver; BPM, *Bambusa piscatorum* McClure*.

The concentration of Si and phytolith in the belowground trunk ranged from 2.30 g kg^-1^ for PPP (*Phyllostachys praecox C. D. Chu‘Prevernalis’*) to 14.07 g kg^-1^ for PHO, and from 5.88 g kg^-1^ for BPM to 14.95 g kg^-1^ for PHO, respectively. The C concentration in phytolith in the rhizome were generally higher than those in the belowground trunk, and both of them varied greatly among the bamboo species. The C concentration in phytolith was highest in PPP (179.99 g kg^-1^), and lowest in BPM (23.44 g kg^-1^). The concentration of PhytOC in dry biomass was also significantly higher in PPP (0.94 g kg^-1^) than the other bamboo species, followed by PHO (0.61 g kg^-1^) and was lowest in PP (0.10 g kg^-1^).

### Estimation of PhytOC Stock of Bamboo in Belowground Biomass

The PhytOC stocks in the rhizome and belowground trunk varied among the bamboo species with the range of 2.30–23.58 and 0.13–3.73 kg ha^-1^, respectively (Table 4). The PhytOC stocks in the rhizome of PP were almost 10 times higher than those in the rhizome of PHO and BPM. The PhytOC stocks in the rhizome were much higher than those in the belowground trunk, accounting for more than 80% of the total belowground biomass across the eight species with the exception of PHO (69.48%). The PhytOC stocks in the aboveground and belowground biomass ranged from 13.00 to 90.36 kg ha^-1^ and from 2.57 to 23.71 kg ha^-1^ among the studied bamboo species, respectively. The PhytOC stocks in the belowground biomass accounted for 5.55–56.76% of the total PhytOC stocks of plant biomass among the eight species, with a mean of higher than 23.36%. The highest proportion was in PPP (56.76%), followed by PP (37.80%). The proportion of PHO and BPM was lowest, with a mean of 7.73 and 5.55%, respectively. According to the distribution area of the bamboo, the total PhytOC stock belowground of the eight species was 9.14 × 10^4^ t, to which 94.84% was contributed by PHMP.

**Table 4:** Estimation of PhytOC stocks via belowground organs of eight monopodial bamboo species in China.

Bamboo species	PhytOC stock of rhizome (kg⋅ha^-1^)	PhytOC stock of belowground trunk (kg⋅ha^-1^)	Belowground PhytOC stock (kg⋅ha^-1^)	Aboveground PhytOC stock (kg⋅ha^-1^)^a^	Plant PhytOC stock (kg⋅ha^-1^)	Ratio of belowground to plant PhytOC stock (%)	Distribution area ( × 10^4^ ha)	Total belowground stock of PhytOC (t)
PHMP	17.11	0.58	17.69	66.18	83.87	21.10	489.78	86657.06
PPP	14.87	2.21	17.07	13.00	30.08	56.76	16.22	2769.23
PP	23.58	0.13	23.71	39.01	62.72	37.80	0.59	139.87
PAMK	8.95	0.20	9.15	56.47	65.62	13.95	4.28	391.82
PGM	22.00	0.43	22.44	90.36	112.80	19.89	4.57	1025.45
PAKK	16.98	3.73	20.72	65.20	85.91	24.11	1.12	232.01
PHO	2.39	1.05	3.44	41.10	44.55	7.73	2.94	101.23
BPM	2.30	0.27	2.57	43.63	46.19	5.55	2.21	56.69
Total	–	–	–	–	–	–	521.71	9.14 × 10^4^


*^*a*^Data were cited from Huang (2014) and Yang (2016). PHMP, *Phyllostachys heterocycla* (Carr.) Mitford ‘Pubescens’; PPP, *Phyllostachys praecox* C. D. Chu ‘Prevernalis’; PP, *Phyllostachys prominens* W. Y. Xiong; PAMK, *Pseudosasa amabilis* (McClure) Keng f; PGM, *Phyllostachys glauca* McClure; PAKK, *Pleioblastus amarus* (Keng) Keng f; PHO, *Phyllostachys heteroclada* Oliver; BPM, *Bambusa piscatorum* McClure.*

## Discussion

The present study showed that the phytolith and PhytOC concentrations in belowground trunk and rhizome differed greatly between bamboo species. Our results were partly similar to the findings by Yang et al. (2015), who found that the phytolith and PhytOC concentrations vary across leaf, branch and culm, and also between bamboo species. However, in comparison with the range of phytolith and PhytOC concentrations in aboveground biomass found by Yang et al. (2015), the quantity of PhytOC in the rhizome and belowground trunk in our study was much smaller. Our study suggested that the PhytOC production capacities of different bamboo species and different organs of the same species vary substantially, which may be ascribed to differences in both physiological properties and the environments. Several studies suggested that the variation of PhytOC concentration in plant depends on the contents of phytolith and C concentration in phytolith, both of which are related to the plant absorption capacities of Si (Ding et al., 2008; Parr et al., 2010). It is well-known that although Si can be taken up by plant roots in the form of Si(OH)_4_ (Gong et al., 2004; Ranganathan et al., 2006), the ability of transpiration for Si varies in bamboos of different species and within different organs (Leng et al., 2009; Li et al., 2014a; Yang et al., 2015). In addition, phylogenetic type, climate, soil, and the efficiency of C encapsulation by the silica are also important factors influencing the absorption and transpiration of Si (Song et al., 2013; Li et al., 2014a,b; Zhang et al., 2017). Among these factors, soil conditions such as water and pH could not only influence the accumulation of soil phytoliths by affecting the stability of soil phytoliths, but also influence plant Si uptake from soil solution by affecting the bioavailability of Si in soils (Parr and Sullivan, 2005; Li et al., 2014c; Yang et al., 2018). For example, plants in soils with low pH and high organic matter are reported to take up and accumulate more Si, and consequently higher PhytOC accumulation (Song et al., 2012b). Liu et al. (2017) found that the contents of Si and PhytOC in the Moso bamboo leaves differed between soils derived from different parent rocks. Similarly, Li et al. (2014c) observed that variation of bioavailable Si of soils developed on different parent rocks could lead to the differences in Si absorption from soil solution and phytolith accumulation in bamboo leaves. Nevertheless, we acknowledge that it is a limitation of our study that the soil properties were not examined, and the mechanisms of Si absorption and phytolith accumulation in belowground trunk and rhizome of bamboo deserves more studies.

In previous studies, the potential of phytolith C bio-sequestration has been largely assessed based on the above- rather than belowground biomass across both agriculture, grassland and forestry ecosystems (Parr et al., 2010; Song et al., 2012b; Li et al., 2013; Ru et al., 2018). One of the important reasons is that the belowground biomass including shoot stumps and roots is usually smaller than the aboveground especially for some Si-accumulator plants, such as sugarcane, rice, and wheat. Another key reason is that some researchers believed that only the aboveground biomass, such as leaves and sheath, can accumulate phytoliths and have high PhytOC concentration (Parr and Sullivan, 2005; Parr et al., 2009; Song et al., 2012b), leading to the potential of belowground C sequestration by phytoliths being overlooked. Our study showed that the belowground trunk and rhizome of bamboo accounted for an average of 39.41% of the total plant biomass, while the belowground material of PP contributed 74.91% of the total weight. These results are consistent with the findings of Wang et al. (2013). The PhytOC stock in the belowground biomass of eight monopodial bamboo species ranged from 2.57 to 23.71 kg ha^-1^, which was comparable to those in the aboveground of grassland (1.64 to 10.36 kg ha^-1^) (Song et al., 2012a), wetland (0.82 to 21 kg ha^-1^) (Li et al., 2013) and wheat (1.64 to 10.36 kg ha^-1^) (Parr and Sullivan, 2011). The PhytOC stock in the belowground biomass of PPP (*Phyllostachys praecox* C. D. Chu ‘Prevernalis’) was even larger than that in its aboveground biomass. These observations suggested that though the PhytOC concentrations in belowground biomass were relatively smaller than those in the leaves or branches of bamboo or in other plants, the large total belowground biomass per hectare of the monopodial bamboo could contribute greatly to the PhytOC stock in belowground. In contrast, the PhytOC stock in the belowground biomass of bamboo species was much lower than that in the aboveground of sugarcane (32.73 to 98.18 kg ha^-1^) (Parr et al., 2009), which could be explained by the higher phytolith accumulation ability and greater biomass of aboveground per unit area of sugarcane compared to monopodial bamboo (Tu, 2011). Qi et al. (2017) observed that the PhytOC stock in belowground biomass was about 40 times of that in aboveground biomass in a typical steppe grassland due to the greater belowground PhytOC content and net primary productivity. In agreement, our study for the first time showed that the PhytOC stock in belowground biomass makes up an average of 23.36% of the total PhytOC stocks of plant biomass among the eight bamboo species, though the percentage value was much smaller than that reported by Qi et al. (2017). Taking the mean value (14.60 kg⋅ha^-1^) of PhytOC stock in belowground biomass across the eight species and China’s current monopodial bamboo area of 5.85 × 10^6^ ha, we estimated that the belowground PhytOC stock of monopodial bamboo is 1.01 × 10^5^ t, and approximately 3.69 × 10^5^ t CO_2_ would be sequestered in belowground phytoliths of Chinese monopodial bamboo forests. According to Huang et al. (2014) and Yang et al. (2015) who estimated that the total aboveground PhytOC stock of the eight species was 4.27 × 10^5^ t, this study further showed that the total belowground PhytOC stock of the eight species was 9.14 × 10^4^ t, accounting for 21.38% of the whole plant PhytOC stock in China. Our study provides an important finding that the belowground biomass of bamboo is a large PhytOC stock that should be taken into account when estimating the potential of PhytOC sequestration of the whole bamboo biomass accurately in future studies. Therefore, the findings here supported our hypothesis that the bamboo species differ greatly in PhytOC concentrations in their belowground biomass and between species, and that the belowground parts make a significant contribution to the total PhytOC sequestration of bamboo plants.

## Conclusion

Our study reveals that the PhytOC concentration in the belowground trunk and rhizome varied among the studied bamboo species. The PhytOC stock in belowground biomass makes up an average of 23.36% of the total PhytOC stocks of plant biomass among the eight bamboo species. Based on our results, approximately 3.69 × 10^5^ t CO_2_ would be sequestered in belowground phytoliths of Chinese monopodial bamboo forests, suggesting that the belowground biomass of bamboo represent a great PhytOC stock, and should not be overlooked in future studies in order to better quantify the PhytOC sequestration capacity.

## Author Contributions

All authors listed have made a substantial, direct and intellectual contribution to the work, and approved it for publication.

## Conflict of Interest Statement

The authors declare that the research was conducted in the absence of any commercial or financial relationships that could be construed as a potential conflict of interest.
